# Surgical videos on the internet: Is this a reliable pedagogical tool in residency training?

**DOI:** 10.1051/sicotj/2022039

**Published:** 2022-09-22

**Authors:** Abdelhamid Ghersi, Jad Mansour, Philippe Marchand, Abdallah Al Rubaie, Pascal Kouyoumdjian, Remy Coulomb

**Affiliations:** 1 Centre Hospitalo-universitaire de Nîmes Rue du Pr. Robert Debré 30029 Nîmes France; 2 Université Montpellier 1 2 Rue de l’École de Médecine 34090 Montpellier France; 3 Laboratoire de Mécanique et Génie Civile (LMGC), CNRS-UM1 860 Rue de St – Priest 34090 Montpellier France

**Keywords:** Femoroacetabular impingement, Hip arthroscopy, Video assessment tool, Guidelines, Surgical training

## Abstract

*Introduction*: Several open access platforms are currently available to help facilitate this online learning; however, whether the platforms are generalized or specialized, peers do not evaluate videos, and they may teach unverified techniques. The purpose of this study was to compare the quality of each website’s content using a specific measurement, analyzing the pedagogical quality of Femoroacetabular impingement (FAI) arthroscopic videos on YouTube versus VuMedi. *Methods*: A prospective study analyzing 20 arthroscopy videos of arthroscopic FAI surgery on two hosting platforms online: 10 on the generalist platform YouTube and 10 on a specialized platform VuMedi. *Results*: The average length of the YouTube videos was 503 ± 355.02 s (198–1430). The average number of views for the YouTube videos was 56,114.6 ± 116,832.61 (1149–383,694). The average length of the VuMedi videos was 797.5 ± 522.5 s (185–1927). The average number of views for the VuMedi video was 10,404.7 ± 10,071.2 (1625–37,115) The average LAP-VEGaS scores of the YouTube and VuMedi videos were 8.2 ± 3.47 (3–15) and 11.95 ± 2.64 (6–15), respectively (*p* < 0.0005). *Discussion*: The use of a specialized website makes it possible to obtain educational videos of better quality. Health professionals need to be aware of this when using it as a resource for learning. Therefore, it should be in the interest of educational institutions to participate in the sharing of videos on this platform or to create their own platform to improve the quality of the information provided and the overall pedagogical experience.

Level of evidence: II

## Introduction

Femoroacetabular impingement (FAI) is a medical entity that has been historically known since 1936, and its definition was only recently updated by Ganz in 2003 following the development of hip arthroscopy [1, 2]. Given this recent development of a multidisciplinary international consensus, “The Warwick Agreement,” along with the increasing number of patients, traditional academic training remains low for this very specialized theme, while the number of media available for online training is increasing [3, 4].

Continuing medical education (CME) and resident learning are changing with the new rules of the health system modernization [5]. Video platforms are currently at the student’s fingertips and are playing a growing role for increasingly overburdened residents [5, 6]. Taking advantage of its accessibility, e-learning has become an increasingly attractive medical training method. The use of virtual simulation and videos before performing real procedures could potentially serve as an excellent educational tool before the actual procedures [7, 8]. In a randomized controlled trial, blinded by an evaluator, a group of medical students who received video instruction significantly improved their venipuncture performance as measured by the checklist, more than the group of students who did not receive video instruction, with scores of 14.15 and 9.18, respectively, out of a total of 18 points [9].

In our modern world, several open access platforms are available, with over four billion videos available. YouTube (https://www.youtube.com) is considered the second most popular website in the world, following Google (https://www.google.com) [10]. In addition, more than 65,000 new videos are uploaded daily [10, 11]. The advantages of this website are its accessibility worldwide and the ability to share medical content easily. However, YouTube is a mainstream generalized website fed by consumers without regulations specific to the health environment, which inevitably includes the risk of disseminating inappropriate information. This led to the establishment of more specialized video platforms dedicated to medical training and information, such as VuMedi (https://www.VuMedi.com): the first network for video education for doctors. This platform aims at helping doctors optimize patient care decision-making by providing authentic and complete education videos from various institutions and practitioners.

However, these two platforms are neither peer-reviewed nor professionally verified techniques. This study hypothesized that a specialized platform presented videos of higher educational merit than a generalist platform.

### Purpose of the study

This study compares the quality of each website contents using a specific measurement, analyzing the pedagogical quality of FAI arthroscopic videos on YouTube versus VuMedi.

## Material and methods

### Study design

A prospective study was conducted between January and March of 2021. Twenty arthroscopy videos of arthroscopic FAI surgery were analyzed on two different online hosting platforms: 10 on the generalist platform YouTube and 10 on a specialized platform, VuMedi. Two surgeons made the initial selection, a junior surgeon and a senior consultant practicing hip arthroscopy. The study was not subject to institutional review by our ethics committee because it did not involve the use of personal data.

### Video selection and exclusion criteria

The selection objective was to choose 10 videos on YouTube and ten on VuMedi. Only one observer made the selection using the following keywords, “FEMOROACETABULAR IMPINGEMENT” or “FAI,” in the search. Before videos were selected, they were sorted by those most viewed. Regarding the YouTube platform, the keyword addition of “HIP ARTHROSCOPY” was required to obtain the ten videos. The following exclusion criteria were used for the videos: incomplete videos, arthroscopies not treating FAI, open surgery, too short, missing video or webinar, FAI rehabilitation, FAI clinic or imaging, patient information included, non-English, and duplicates. The results were deliberately limited to ten videos per platform to simulate a practical search of a junior user, not a systematic search.

### Judgment criteria

Two surgeons independently evaluated each video. For each video, the length and number of views at the time of analysis were recorded. The 10 videos in each group were analyzed and evaluated according to two scoring standards.

**Figure 1:**
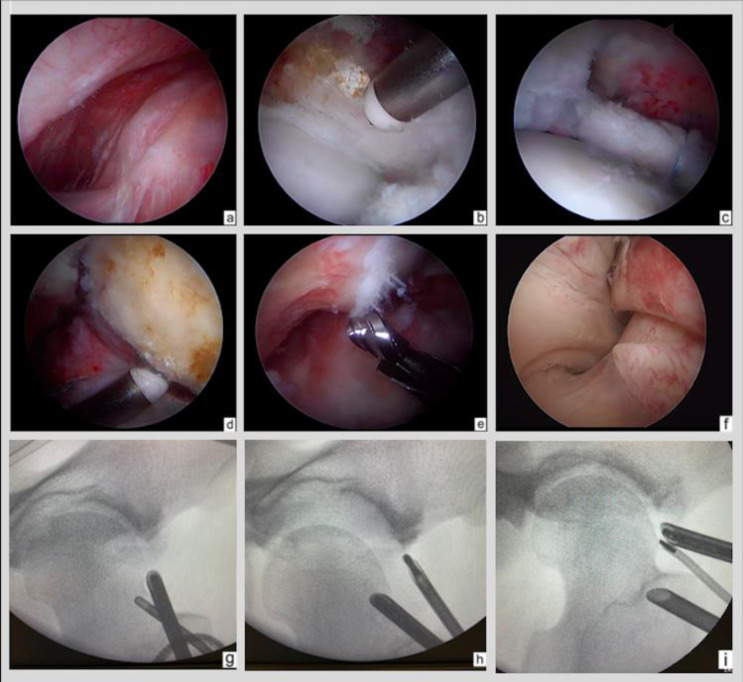
Graded surgical steps. a: Locating the medial plica, b: Acetabular step – exposure of the acetabular clamp, c: Labrum suture and “suction cup effect”, d: Femoral step – exposure of the femoral cam, e: Femoral step – control of the femoral resection, f: Locating the vessel, g: Preoperative imaging of the forceps, h: Checking clamp resection, i: Preoperative imaging of the cam.

The first LAP-VEGaS-FAI is an adaptation of the LAP-VEGaS score, which judges the character of the pedagogical merit of the endoscopic surgical videos [12]. The LAP-VEGaS score was used to analyze the videos according to the following criteria: author information/conflicts of interest, presentation of the case, surgical installation, surgical approach, description of procedure, anatomical description, postoperative results, tips and tricks, audio comments and/or written in English, and video quality. The primary outcome of this study was based on the 18-point score of the LAP-VEGaS-FAI for each video, with the minimum being 0 points. The LAP-VEGaS and LAP-VEGaS-FAI scores are presented in Table 1. Figure 1 specifies the arthroscopic key points that made it possible to adapt the score to fit the management of femoroacetabular impingement.

**Table 1:** LAP-VEGaS Video Assessment Tool (original in bold)/LAP-VEGaS-FAI (modified in italics).

Item description	Step presented (0)	Presented partly (+1)	Presented completely (+2)
**1. Information on authors and institutions. Video title, including name of the procedure and pathology treated**	□	□	□
*– Authors + conflict of interest + title = + 2*			
*– If missing 1 = + 1*			
*– If missing 2 = 0*			
**2. Formal presentation of the case, including clinical details and imaging of the patient, the indication for surgery. The anonymity of the patient is preserved**	□	□	□
*– Presentation case = clinical + imaging + anonymity = + 2*			
*– If missing 1 = + 1*			
*– If no presentation of the case = 0*			
**3. Patient position, first and organization surgical team**	□	□	□
– *Position + VA (capsulotomy) + organization = + 2*			
*– If missing 1 = + 1*			
– *If missing 2 = 0*			
**4. The surgical procedure is presented in a standardized way, step by step**	□	□	□
– *Bone gesture + labral gesture/cartilaginous = + 2*			
– *If missing 1 = + 1*			
– *If missing 2 = 0*			
**5. Intraoperative results are clearly demonstrated, with a constant reference to anatomy**	□	□	□
– *Central explo (clamp – labrum – cartilage; wave sign) + periph (cam) = + 2*			
*– If missing 1 = + 1*			
*– If missing 2 = 0*			
**6. Postoperative results of the procedure are presented**	□	□	□
*– Postoperative Rx + labrum sucking effect*			
*+ dynamic testing = + 2*			
*– If missing 1 = + 1*			
*– If missing 2 = 0*			
**7. Additional graphic aids are included, such as diagrams, snapshots, and photos, to show anatomical landmarks, discoveries relevant or unexpected, or to present additional educational content (scopy re-e time)**	□	□	□
*– Tips and tricks + scopy (milling acetabulum + femur) + educational content additional (plica/vx) = + 2*			
*– If missing 1 = + 1*			
*– If missing 2 = 0*			
**8. An audio/written commentary in English is provided**	□	□	□
*– Audio/written commentary = + 2*			
*– Incomplete/poor quality = + 1*			
*– If missing 2 = 0*			
**9. Image quality is appropriate with a clear and constant view of the operating field. Video is smooth with proper speed (hand vision)**	□	□	□
*– Excellent image quality + operative field vision = + 2*			
*– If quality is average or absence of operative field vision = + 1*			
*– If poor quality = 0*			

The second score allowed the evaluation of the perceived quality of the video. The reviewers were asked if they would have recommended the video to a peer or resident, and they were also asked if they would have accepted the video for publication and/or a podium presentation, using closed-ended questions and a 5-point Likert scale. Table 2 shows the 5-point Likert scale.

**Table 2:** Five-point Likert scale on single-peer/trainer video recommendations and the acceptance of the video for publication or presentation on the podium.

Items	1	2	3	4	5
1. I recommend this video to a peer/intern.	□ strongly disagree	□ disagree	□ neither agree nor disagree	□ agree	□ strongly agree
2. The video is of satisfactory quality for a presentation/publication.	□ strongly disagree	□ disagree	□ neither agree nor disagree	□ agree	□ strongly agree
3. Overall video quality	□ very poor	□ poor	□average	□ good	□ very good
4. Overall educational content of the video.	□ very poor	□ poor	□ average	□ good	□ very good
5. Time required to complete the correction note (only the time needed to complete the note, not the time of the video).	□ >4 min	□ 3–4 min	□ 2–3 min	□ 1–2 min	□ <1 min
6. How satisfied are you with the use of the score?	□ very unsatisfied	□ unsatisfied	□ neither unsatisfied nor satisfied	□ satisfied	□ very satisfied
7. I would like you to use the new scoring system of LAP-VEGaS?	□ strongly disagree	□ disagree	□ neither agree nor disagree	□ agree	□ strongly agree
8. The elements of the LAP-VEGaS score help to differentiate educational/non-educational videos of good or poor quality.	□ strongly disagree	□ disagree	□ neither agree nor disagree	□ agree	□ strongly agree

### Statistics

The statistics were calculated using IBM^©^ SPSS^©^ Statistics software, version 23.0.0.0. Quantitative variables were presented by mean, standard deviations, minimum, and maximum values. Qualitative variables were matched with their number and percentage. Unmatched quantitative variables were compared using a student test, and the matched variables were compared using a Wilcoxon test. Qualitative variables were compared using a χ^2^-test. Intra-class correlation coefficients were calculated to define inter- and intra-observer variability for all measured parameters. A difference was considered statistically significant when the degree of significance was less than or equal to 0.05.

## Results

### Search results

**Figure 2:**
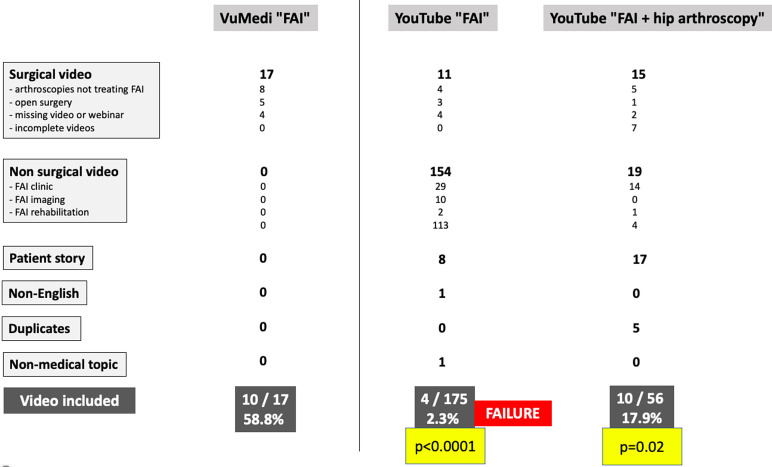
Video search flowchart.

The search results for “FEMOROACETABULAR IMPINGEMENT” or “FAI” on VuMedi and YouTube were presented. In the YouTube group, given the search’s failure to find 10 videos with these keywords alone, the search was refined using the keywords “FAI” + “HIP ARTHROSCOPY.” The research was statistically more cost-effective on the VuMedi platform versus YouTube, according to the first modality (*p* < 0.0001) and the second research modality (*p* = 0.02) (Figure 2).

### Video features

The average length of the YouTube videos was 503 ± 355.02 s (198–1430). The average number of views for the YouTube videos was 56,114.6 ± 116,832.61 (1149–383,694). The average length of the VuMedi videos was 797.5 ± 522.5 s (185–1927). The average number of views for the VuMedi video was 10,404.7 ± 10,071.2 (1625–37,115). Despite an average of five times more views in the YouTube group and an average duration of 1.5 times longer in the VuMedi group, there was no statistical difference between the two groups regarding the duration (*p* = 0.16) and the number of views (*p* = 0.25).

### Inter-observer correlation

The intraclass correlation coefficient between observers 1 and 2 for the LAP-VEGaS scores was 0.951 for the YouTube videos and 0.878 for the VuMedi videos. The coefficient of the overall intraclass correlation between observers 1 and 2 for LAP-VEGaS scores was 0.939.

Regarding the Likert scale, the intra-class correlation coefficient between observers 1 and 2 was 0.569 for the YouTube videos and 0.503 for the VuMedi videos. The coefficient of the global intraclass correlation between observers 1 and 2 for the Likert scale was 0.604.

The correlation between the two reviewers appeared to be better when using the LAP-VEGaS score.

### Video evaluation

**Figure 3:**
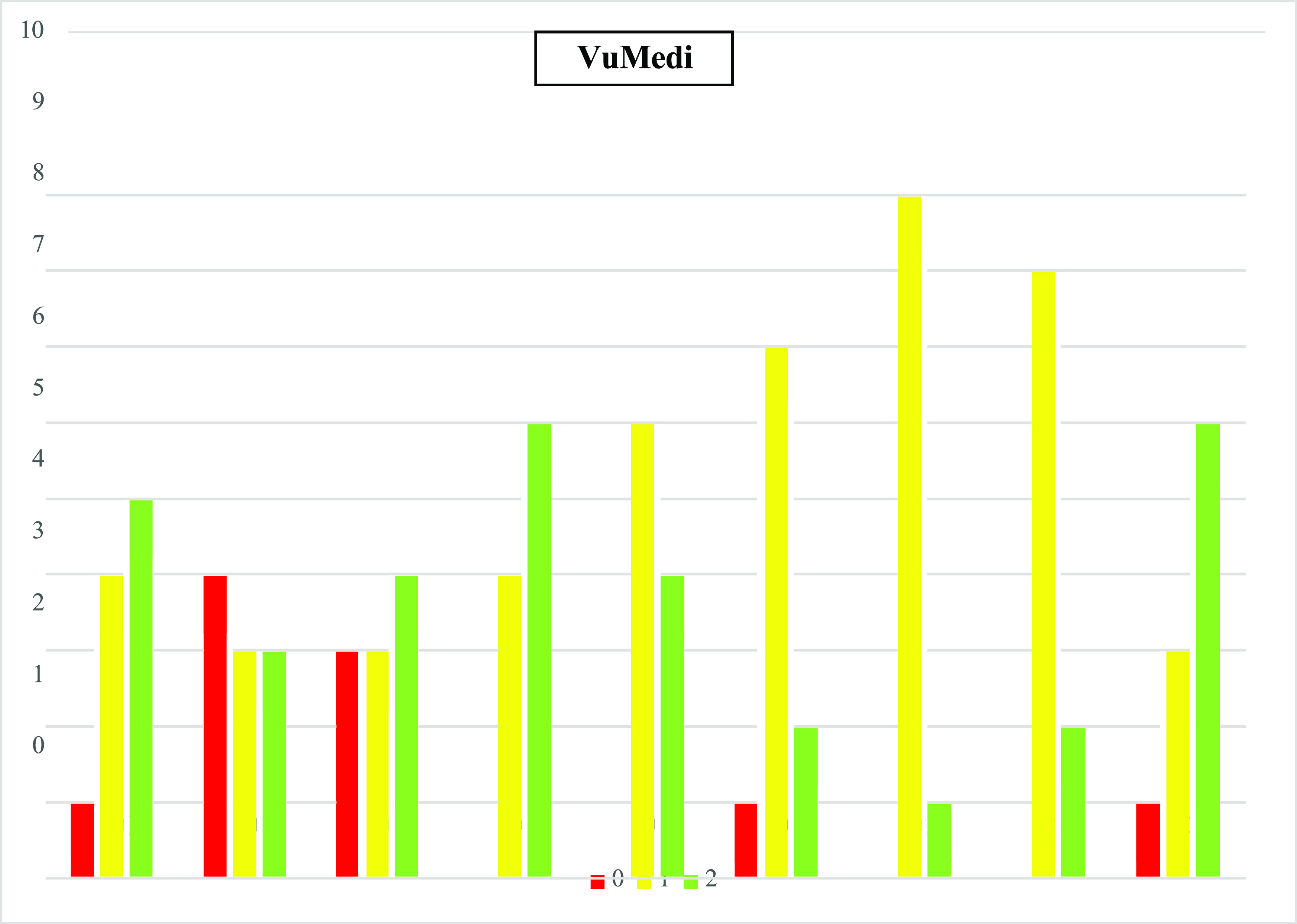
Distribution of scores for each of the nine items of the VuMedi videos. Q1-Q9: Items from the assessment tool: 0 – Item not shown; 1 – Item partially presented; and 2 – Widely featured item.

**Figure 4:**
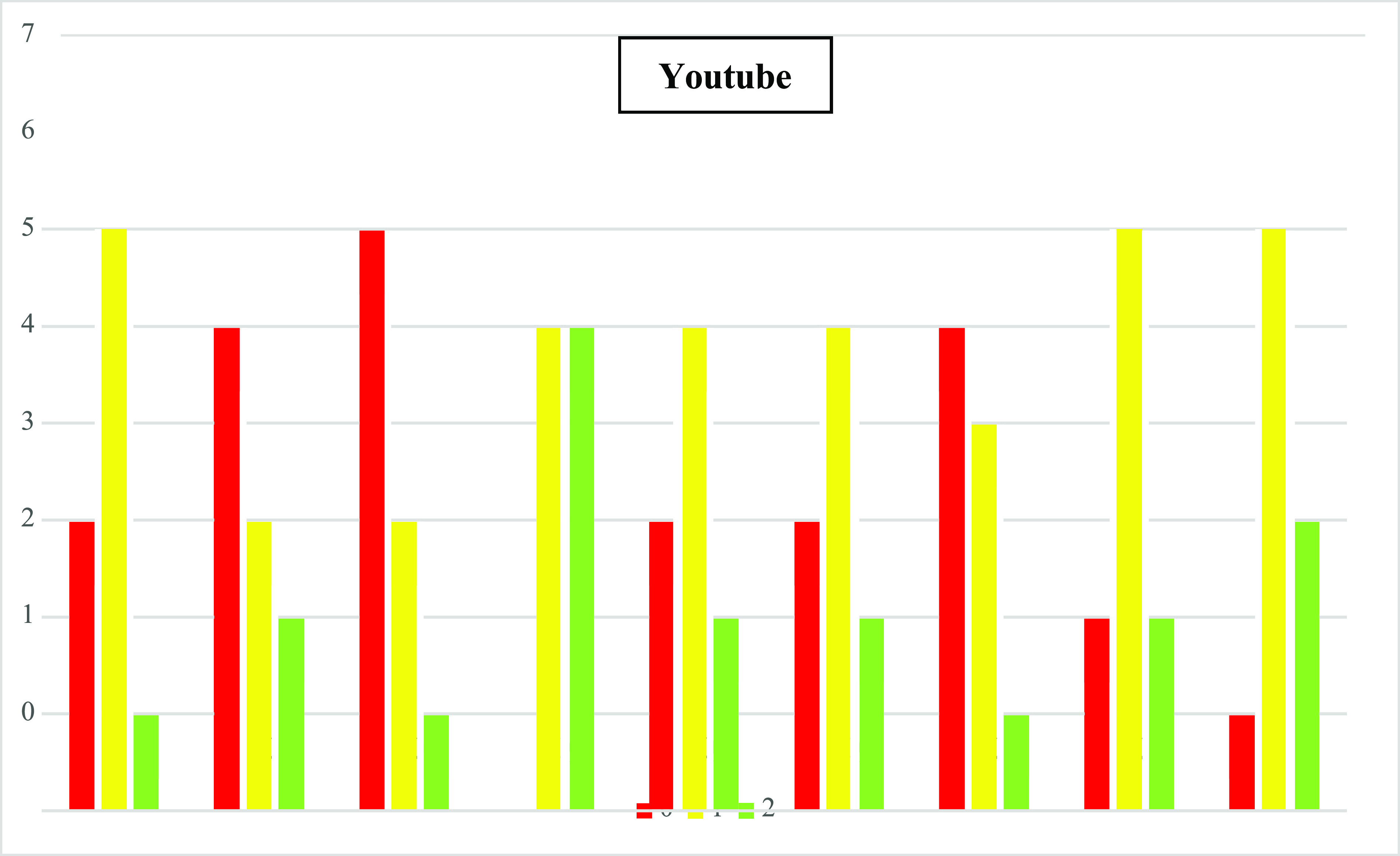
Distribution of scores for each of the nine items of the VuMedi videos. Q1–Q9: Items from the assessment tool: 0 – Item not shown; 1 – Item partially presented; and 2 – Widely featured item.

The average LAP-VEGaS scores of the YouTube and VuMedi videos were 8.2 ± 3.47 (3–15) and 11.95 ± 2.64 (6–15), respectively (*p* < 0.0005). Figures 3 and 4 represent the distribution of the scores for each of the nine items in the evaluated video.

**Figure 5:**
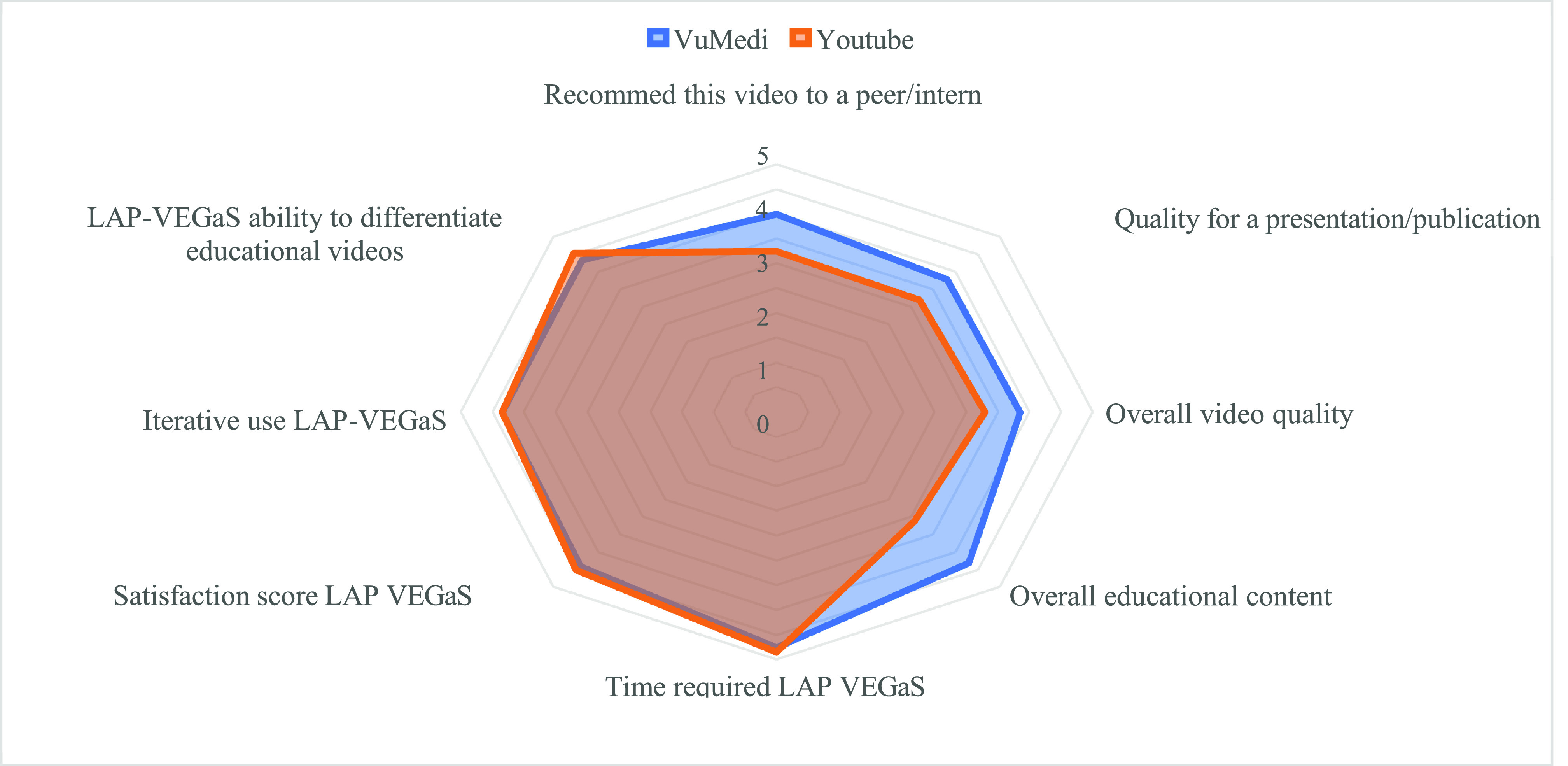
Comparative diagram of the 5-point Likert scale.

The evaluators indicated that the average time to complete the LAP-VEGaS-FAI score was less than 1 min. In addition, the evaluators were very satisfied with the use of the tool. The LAP-VEGaS video evaluation assigned average scores of 4.45 and 4.35, respectively, to the questions regarding “general satisfaction” and “probability of iterative use” on the 5-point Likert scale (Figure 5).

## Discussion

This study shows a significant difference between the educational value of videos from a general platform (YouTube) against a specialized platform (VuMedi). The average score obtained by the LAP-VEGaS-FAI rating was 8.2 for YouTube, compared to VuMedi’s 11.95. Despite a lower viewership (10,404.7 vs. 56,114.6 average views), the specialized platform is more beneficial to surgical pedagogy.

The advent of information and communication technologies can potentially improve surgical education. We found that video learning is currently a feature of surgical preparation for residents and surgeons. A thriving diffusion presents the opportunity to account for the current flaws in surgical education. However, many ethical entities could be breached while building those surgical videos. Dorfman et al. reported in a systematic review that sharing live videos or patients’ photos in plastic surgery can have significant ethical implications, and particular guidelines should be used [13]. In addition, Turnbull and Emsley reported the ethical and legal considerations in a video recording of ophthalmic surgery and considered that legal systems should be placed to deal with storage issues, consent forms, and particular requests [14].

The LAP-VEGaS-FAI score comes from the LAP-VEGAS score and is conditioned for this study to evaluate videos that treat femoroacetabular impingement [12]. Prior to the establishment of LAP-VEGaS guidelines, there was no accreditation or regulation for the use of medical videos as training resources [15]. The LAP-VEGaS video assessment tool aims to facilitate and standardize the peer review process of submitted videos. However, this score has limitations because this score is generic and must be adapted to fit the pathology of interest. Some original guidelines may not be relevant for certain procedures. In addition, many online videos focus on a specific procedure aspect, displaying the technical nuances or variations of established operations. These videos can be of very good quality while still having a bad evaluation score.

Finally, teamwork and communication remain essential qualities for safe and effective performance. They have not been explored in this video assessment tool, which focuses on the technical skills of the surgeon [16]. In addition, although the use of videos for surgical preparation is customary nowadays, the quality of the available videos is not assured, and no provision was made for evaluating the technical quality of the surgical content [17, 18]. Several studies have addressed this topic and evaluated the quality of YouTube in relation to medical procedures and concluded that this platform offers enormous variability in quality [17–19]. For this reason, some authors suggest that the solution could be creating a ranking system distinguishing between videos and implementing a quality assurance system that is updated regularly [20, 21].

Among the other limitations, each video was only analyzed twice for simplicity. The study by Celentano et al. reported identical score reliability between two or three assessors [12]. On the other hand, the presence of an evaluator who was not specialized in hip arthroscopy could have affected the quality of the analysis. However, as the study assesses compliance with LAP-VEGaS guidelines rather than an analysis of the surgical technique, we judged the level of expertise of the evaluators as a secondary element. Finally, the non-systematic nature of the search was too narrow, which may have excluded other relevant videos in each group.

This study has a strong potential to be one of the few articles in the field of orthopedic surgery to be interested in surgical educational videos. This can lead to a second comparative study that will assess and compare the quality of training between traditional academic teaching and video teaching.

Many surgically treated femoroacetabular impingement videos analyzed in this study did not demonstrate high-quality content when applying the LAP-VEGaS criteria. Using a specialized website makes it possible to obtain better quality and content educational videos. Health professionals need to be aware of this difference when using random surgical videos as a resource for learning. Therefore, it should be in the interest of educational institutions to share optimal and complete surgical videos on those platforms and/ or to create their own platform to improve the quality of the information provided and the overall pedagogical experience.
