# Correction Notice to: Surgical videos on the internet: Is this a reliable pedagogical tool in residency training?

**DOI:** 10.1051/sicotj/2022047

**Published:** 2022-12-16

**Authors:** Abdelhamid Ghersi, Jad Mansour, Philippe Marchand, Abdullah Alrubaie, Pascal Kouyoumdjian, Remy Coulomb

**Affiliations:** 1 Centre Hospitalo-universitaire de Nîmes Rue du Pr. Robert Debré 30029 Nîmes France; 2 Université Montpellier 1 2 Rue de l’École de Médecine 34090 Montpellier France; 3 Laboratoire de Mécanique et Génie Civile (LMGC), CNRS-UM1 860 Rue de St – Priest 34090 Montpellier France

The article was published September 22, 2022 with an error in the name of one of the co-author.

The full name of this co-author is: Abdullah Alrubaie.

